# What Should Be Taught in Secondary Schools’ Nutrition and Food Systems Education? Views from Prominent Food-Related Professionals in Australia

**DOI:** 10.3390/nu9111207

**Published:** 2017-11-02

**Authors:** Sanaz Sadegholvad, Heather Yeatman, Anne-Maree Parrish, Anthony Worsley

**Affiliations:** 1 School of Health and Society, Faculty of Social Sciences, University of Wollongong, Wollongong, NSW 2522, Australia; hyeatman@uow.edu.au (H.Y.); aparrish@uow.edu.au (A.-M.P.); 2 Centre for Physical Activity Research, School of Exercise and Nutrition Sciences, Deakin University, Burwood, VIC 3125, Australia; anthony.worsley@deakin.edu.au

**Keywords:** nutrition, food systems, education, secondary schools, and adolescents

## Abstract

Education can help young people to attain the knowledge and the skills that they need to make proper food choices and develop lifelong healthy eating patterns. This study explored the perspectives of prominent food-related professionals in Australia regarding essential nutrition and food systems (N&FS) education programs for adolescents during formal education. Semi-structured interviews were conducted with 21 prominent food-related professionals in Australia. Interview transcripts were analysed thematically. Four essential areas for N&FS education programs were identified. (1) Key nutrition messages to a healthy lifestyle; (2) Skill development programs to enhance health and wellbeing; (3) Ethical food-related lessons to support environmental sustainability, farm animal welfare, local producers, and food security; and, (4) Introductory lessons about foods from farm to plate to facilitate more informed food choices. Findings of this study may provide new insights for curriculum developers in Australia for further assessment of the current gaps in N&FS components of secondary school curriculum. Integration of these four areas into secondary school curricula has the potential to enhance adolescents’ knowledge of important scientific and ethical issues in a range of N&FS fields, and enable them to develop fundamental food-related life skills that are supportive of health and wellbeing.

## 1. Introduction

Globally, unhealthy dietary patterns and sedentary behaviours are known as leading risk factors for health and wellbeing. Rapid urbanisation, changing lifestyles, and increased the production and ready availability of highly processed foods have shifted people’s dietary patterns. This dietary has increased the consumption of fats, energy-dense foods, and for many people, the inadequate consumption of vegetables, fruits, and whole grains [1]. The Australian population does not consume recommended amounts of different food groups [2]. Less than four percent consume the recommended amounts of vegetables, legumes, and beans; only 30% meet the recommendation for fruits; around 30% meet the recommendation for grain; and approximately 35% of the total daily energy intake of Australians comes from discretionary foods, like sugar sweetened beverages, cakes, and confectionery [2].

Dietary behaviours during adolescence contribute to the establishment of lifelong eating patterns [3]. Unfortunately, the dietary behaviours of a large proportion of Australian adolescents deviate from the national dietary recommendations [4] and increase their risks of nutrition-related health problems, like obesity and insulin resistance [4].

Healthy eating habit among adolescents and children are essential for healthy growth, cognitive development and other aspects of health and wellbeing [5]. In addition, adaption of healthy eating habit has been linked to reduced risk of chronic diseases in their future life [5].

As Story et al. note: “Nutrition education can help young people attain the knowledge and skills they need to make healthful food choices and develop lifelong healthy eating patterns. Schools are ideal settings for nutrition education because they reach most youth, and nutrition fits into several subject areas including health, science, and consumer science” [6] (p. 121).

School-based nutrition education curricula should aim to improve students’ knowledge, skills, self-efficacy, and behaviour aligned with the dietary guidelines [7]. However, schools in many western [7,8,9] and non-western countries [10] have not used their potential to deliver food-related education programs [7,8,9,10] a review study of adolescents’ food literacy programs [11] identified lack of effective programs for adolescents, and uncovered the greater focus of current programs to educate younger adolescents rather than older adolescents [11].

Globally, the major focus of current school-based education programs has been on promoting healthy dietary behaviours [12,13,14,15], and Australian investigators have also shown a similar trend in their studies [16,17,18]. However, some nutrition researchers have argued that nutrition education programs should not be limited to health-related aspects of food consumption [19,20,21,22,23,24]. These educators highlighted the need to educate students [19,22,24,25], or more broadly consumers, about food systems-related issues [21,23,26], covering production, processing, packaging, distribution, retailing, consumption, and wastage of food [27]. A major reason for this emphasis on the different aspects of food systems is to promote informed food choices that aim to sustain and protect the environment.

It is well-established that food production and consumption impact the environmental sustainability [23,28,29]. For example, current agricultural practices are contributing to greenhouse gas emissions, excess water use and land degradation [27,30,31]. Consequently, the dietary patterns and food choices that are more supportive of environmental sustainability are recommended [27,28]. Australians’ current eating patterns pose risks to both environmental sustainability and people’s health [27]. For example, in a very recent literature the problem of excess energy intake in western diets, including Australia is reported. In this literature it is highlighted that discretionary foods not only contribute to increased energy intake while lack the nutrients, but also “inflate environmental burdens of diets” [32].

Western and non-western studies have reported the importance of food systems knowledge and relevant education programs to make more informed food choices [19,21,24]. However, previous researches have not shown significant attention toward school-based food systems education programs. The majority of nutrition education programs within the school setting mainly aim to promote health-related aspects of dietary behaviours (e.g., [33,34,35]).

The importance of nutrition and food systems (N&FS) education has been highlighted by several studies as a mechanism to improve adolescents’ capacities to make more informed food choices [19,22,24,25]. Education about specific areas of the N&FS have been highlighted in the literature. For example, education on food groups and dietary guidelines to support adolescents in their selection of healthier food options and healthy eating patterns [24]. Awareness of the association between foods and the environment to promote food choices that are more supportive of environmental sustainability [22,24,25]. Education about the welfare of farm animals has been raised in literature to promote selection of animal-welfare friendly food products [22]. In addition, the importance of informing adolescents about the consequences of their food choices on local farmers has been highlighted [25].

Australian adolescent’s knowledge of some aspects of N&FS was explored in a recent study among 131 secondary school students aged 12–17 [36]. The study results showed participants’ very limited knowledge about food ethics (environmental sustainability and animal welfare) [36]. Findings of this study also revealed participants’ lack of understanding of information on food packages, which negatively affected the adolescents’ willingness to read food labels [36]. These findings are consistent with another study related to the Australia that explored Australian food professionals’ views of Australian adolescents’ knowledge of N&FS [26]. Food professionals in Sadegholvad and colleagues’ study perceived that Australian adolescents, have insufficient knowledge of N&FS issues and knowledge shortage was particularly highlighted in relation to food production processes in conjunction with food ethics [26].

In the current curriculum for the secondary schools in Australia, there are two key areas where food and nutrition topics are covered, to some extent including ‘Health and Physical Education’ and ‘Design and Technologies’ [37]. However, it is unlikely that different components on N&FS, such as those relating to the connections among people’s food choices, food production, and food ethics (e.g., environmental sustainability) are well addressed. In addition, it is not known what, if any, research informed of the development of these curriculum areas. Also, the actual average hours currently allocated for N&FS education in different years in secondary schools across the Australia is not known. The qualitative study reported here aimed to focus on one part of the current gaps in literature related to the appropriate N&FS curriculum for secondary schools in Australia. This study addressed the perspectives of the prominent food-related professionals in Australia about essential N&FS components for secondary schools.

## 2. Materials and Methods

A group of prominent food-related professionals were recruited who had expertise in particular area(s) of nutrition and food systems (N&FS). The participants included nutritionists, dietitians, public health nutritionists, home economists, environmental scientists, agricultural scientists, and veterinary scientists (experts in animal welfare and farm animal production systems). Purposive sampling was used to select highly experienced professionals from each food-related discipline, [38]. The participants included: academics in top ranked universities of Australia; professionals who held key roles in well-recognised government and non-government organisations; as well as senior community nutrition educators/promoters in Australia. For this purpose a thorough investigation of the websites of all the relevant schools or departments related to major universities across Australia was performed. The profiles of academic staff were explored to identify the potential academic participants. A thorough web-based investigation of relevant government and non-government organisations in Australia was undertaken to select a group of non-academic professionals. A final list of forty potential participants was developed and confirmed by all of the authors of this study.

Ethics approval was provided from the Human Research Ethics Committee (Health and Medical) of the University of Wollongong (approval No.: HE12/277). Potential participants were invited via email. The professionals who accepted the invitation, received electronic versions of the information sheet, interview questions, and a consent form, one to two week(s) before each interview. Dates, times, and locations for all of the interviews were arranged via email.

All of the participants provided recorded verbal and/or written consent before and/or at the beginning of their interviews. Semi-structured face-to face (*n* = 7) or telephone–based (*n* = 14) interviews were conducted between August 2012 and March 2014 to explore the professionals’ views of the essential components of education programs for secondary school students. The extended period of data collection reflected limited availabilities.

Very broad interview questions were used to minimise leading the conversations and to provide the opportunity for participants to refer to those parts of nutrition and food systems that were related to their area(s) of proficiency. Interview questions were designed by the authors of this study and were confirmed by four experienced nutrition-related academics at the University of Wollongong, who were experienced in conduction qualitative research.

Prior to this study, the interview questions were used in a similar study for interviewing 28 food-relate professionals in Iran [24].

Two key open-ended questions and relevant prompts (if necessary) were used in all of the interviews. The key questions were:

(1) What are the important nutrition and food systems (N&FS) knowledge issues for Australian school-leavers? Why?
(2) Of the important N&FS education programs for high school students identified, which ones would you consider to be the most important? Why?

The participants were asked to focus on those areas of N&FS that were directly linked to their area(s) of expertise.

Simultaneous data collection and data analysis were performed to continue the interviews until saturation occurred [39]. Thematic saturation occurred after interviewing twenty-one participants [39]. The interviewed professionals included: four dietitians, four home economists, four nutritionists, three public health nutritionists, one public health expert, two food scientists, one environmental scientist, and two experts in animal welfare and animal-sourced food production systems.

Audio-recorded interviews were transcribed verbatim by a professional audio transcriber and crosschecked by the first author. Interview transcripts were analysed thematically [40] using NVivo 10, a qualitative data analysis package. Inductive thematic analysis was used resulting in the identified themes being strongly linked to the data themselves [40,41]. The data set was coded without using any pre-existing coding frame or analytic preconceptions, and the process of data analysis was completely data driven [40]. The analysis method undertaken included: reading and re-reading the interview transcripts; generating initial codes; identifying potential themes; reviewing themes; defining and naming themes; and, developing report [40]. In addition to the first analysis of the dataset, a second manual analysis of all the interview transcripts was performed to identify any potential new theme that was missed over the first analysis. Minor changes occurred after the second analysis. Identified themes were reviewed and areas of N&FS education were developed, named, and confirmed.

## 3. Results

The essential components for nutrition and food systems (N&FS) education programs for secondary school students identified by the participants were grouped into four areas. These areas were: (1) Key nutrition to support a healthy lifestyle; (2) skill development to enhance health and wellbeing; (3) ethical food-related components to support environmental sustainability, farm animal welfare, local producers, and food security; and, (4) introductory components about foods from farm to plate to make more informed food choices. Each of these areas is described separately below.

### 3.1. Key Nutrition Messages to Support a Healthy Lifestyle

The majority of the participants across the different groups of professionals referred to the basic recommendations of national policy guides as one of the most important components for school-based education programs. The aim was to develop a ‘basic understanding’ of healthy dietary recommendations among students. They believed that the ‘core food groups’ should be introduced as the ‘building blocks’ of nutrition education for the formal education system in Australia.

Some of the participants highlighted that the focus needed to be on ‘basic’ and ‘simple’ messages. They explained that it was not necessary for adolescents to know the details of the current dietary guidelines, but it was necessary for them to know “what are the core food groups”, “roughly how much they need to eat to meet their health requirements”, and “the equivalents” in each group. In this area some participants reported that adolescents need to know that they should consume ‘plenty’ of vegetables and legumes, ‘balanced’ amounts of cereals and fruits, ‘smaller’ amounts of animal-based products, and ‘a little’ amounts of healthy oils on a daily basis. They also reported the need to raise students’ awareness of the relationships between each food group and people’s health to encourage them “to consume a variety of all core food groups in balance”.

“I think they should have at least basic knowledge of food groups and what the major nutrients sources are. So I don’t think they need to even understand completely why they need all different foods but at least have some concept of variety and, and what might be important in variety and, and probably an understanding of the core food groups so food groups that they actually may get nutrition from as opposed to a lot of the other things that they might be eating.”

—Dietitian

Two of the dietitians and one of the public health nutritionists underscored the important role of a “positive push” in nutrition education programs for children and adolescents. They considered that the nutrition-related messages needed to shift toward more positive and encouraging messages rather than negative and prohibiting ones.

“I think there needs to be a real positive push towards nutrition and health so that, not just always focusing on the negative that fats are bad, and too much energy is bad, and too many soft drinks are bad, often that’s the message kids get rather than you know what does a healthy nutritious diet look like and, and why is it important to be able to understand that.”

—Public health nutritionist

Some of the public health nutritionists, dietitians, nutritionists, and home economists also identified three other nutrition education issues for secondary school students. One was related to the importance of maintaining healthy weight through appropriate diet and physical activity without developing a negative body image. The second issue related to nutrition requirements during pregnancy, as there was the potential for adolescent pregnancy. The last highlighted issue was related to providing encouraging messages about the importance of breastfeeding to create a ‘positive attitude’ among Australian adolescents about this issue. Nutrition education in relation to pregnancy and breastfeeding was recommended for students who were studying in the last two years of secondary school.

“The average school-leaver needs to understand the needs to support a healthy pregnancy… They should consider the whole issue of breastfeeding. …most boys and girls have established their attitude toward breastfeeding by the time of 15 and 16. So young age group needs to be given some facts and information about the value and place of breastfeeding.”

—Nutritionist

Other participants commented on the importance of avoiding unnecessary information, using the example of nutrition for early parenthood. They believed it was more appropriate to provide this kind of information at more relevant stages of life (e.g., “when a person becomes pregnant”).

### 3.2. Skills Development Programs to Enhance Health and Wellbeing

Most of the participants, including home economists, public health nutritionists, dietitians, nutritionists, and the public health expert, underscored the need for adolescents to build and develop a range of essential food-related, life skills, supportive of healthy dietary practices in the secondary school. For example, it was noted that students needed to develop enough skills and confidence to enable them to take action for “what are we going to have for dinner tonight?” One of the dietitians expressed the view that the “old home economics education type model” is more useful for everyday life as compared to the “academic-based model of nutrition education” (implying a more science-based and theoretical approach).

These professionals identified a range of necessary skills from food ‘planning’ and ‘selection’, to ‘safe food storage’, and also the skills to grow vegetables. In some cases participants from the different groups of professionals gave specific attention to particular skill(s) (as mentioned in following paragraphs).

Most of the participants mentioned the need to enhance students’ understanding of food labels to make more informed food choices. Some of them also mentioned the need for developing skills to ‘critically interpret’ food labels to avoid being manipulated by the food industry. The public health expert noted that building food label interpretation skills among adolescents and other population groups “pushes the food industry to improve different aspects of their products”.

“They (students) need to understand food labelling and what marketing and branding is to make healthy food choices. They need to be educated in how to guide their way through food packages, labels and supermarket choices, so to be able to compare some of the kinds of food for the best option.”

—Public health nutritionist

Most of the home economists, public health nutritionists and nutritionists reported the need to offer budget management education to secondary school students to enable them to select, shop, and prepare affordable and healthy foods. Some of these professionals referred to a common view among some people in Australia that “it is expensive to eat healthy foods” and that this needed to be corrected. The budget management lessons they recommended should include “the best value seasonal ingredients”; “learn to freeze particular foods when they are cheap”; and, learn to select affordable alternatives (e.g., “canned lentils and beans as a cheap alternative to meat”).

“They need to learn to utilise those convenience foods judiciously not relying totally on frozen foods and canned foods but incorporate them because they often are nutritionally equivalent to fresh foods. For example, buying dried chick peas and soaking them overnight, a canned—alternative can be just as cheap, you can usually buy a can of chick peas or lentils for a dollar, so every now and again, you know if you had those sort of staples in your pantry that’s a really good thing to have on hand.”

—Home economist

In contrast, one of the nutritionists thought that budget management education was unnecessary. She expressed:

“I think that kind of stuff is boring and I, I think it will turn students off if that’s how you want to present food and nutrition is about budgeting on a, on a low budget.”

—Nutritionist

Other essential skill-development programs for students were reported, including: food preparation and cooking skills to provide ‘healthy’, ‘affordable’, ‘quick’, ‘simple’, ‘tasty’, and ‘attractive’ meals; and, food safety skills to ‘prepare’ and ‘keep foods safely’. In this study, food preparation and cooking skills (consistent with the Australian dietary guidelines); and skills to prepare and store foods safely were mentioned most frequently as the food skills that should be included in school education programs. Some of the interviewees explained that if the students are equipped with important textbook-based nutrition knowledge issues without building their food preparation skills, it will not be very useful for their everyday lives. For example, one of the dietitians mentioned that if students know that oily fish contains healthy oil and protein, but if they do not know how to prepare it in a tasty and healthy way, then it is not enough to encourage them to consume it on a regular basis.

“Preparation and presentation of foods for good health and satisfaction to support good and enjoyable eating experiences should be part of school-based education. …Food safety skills go hand-in-hand with cooking skills.”

—Home economist

Lastly, the home economists noted that cooking courses conducted by qualified teachers have the potential to train students about a range of other essential food knowledge issues and relevant skills, such as: food costs; affordable ingredients; recipe reading; principles of safe food preparation; nutrient composition of meals; the importance of key nutrients for adolescents’ health; basic way to combine foods from the core food groups in a meal; and, healthy replacements in each food group.

Seven participants from groups of public health nutritionists, nutritionists, home economists, and a dietitian reported that it was important for schools to provide students with opportunities to develop skills for growing plant-based foods (in schools or any other available settings arranged by schools). Some participants also suggested that students should visit farms to become familiar with growing techniques. School-based gardening activities would encourage students to consume more plant-based products, increase familiarity with ‘farming practices’, and also inform students to consider gardening skills as a budget management strategy. However overall, growing foods was less frequently mentioned as compared to the other skills identified by the participants.

“I think children learn by experience so it would be useful for them to have practical experience in terms of food growing. It can be useful for families with limited resources. Students can grow certain vegetables at school, or visiting farms to understand a little bit about farming practices. I think children just don’t get enough exposure to that particularly kids in the city.”

—Public health nutritionist

### 3.3. Ethical Food-Related Lessons to Support Environmental Sustainability, Farm Animal Welfare, Local Producers and Food Security

Twelve participants (more than half of the participants) from across different groups underscored the need to educate secondary school students about value-based ethical issues that are associated with food production and consumption. They recommended the inclusion of ethics lessons that aimed to promote more informed food choices among young Australians to protect the environment, and farm animal welfare and local farmers, and to reduce food insecurity.

A summary of the ethical issues raised by the interviewees is presented in Table 1. All of the key food-related ethical issues presented in the Table 1 have same level of importance. In fact, the participant did not present a hierarchy of the most important ethical food-related issues for adolescents.

The professionals, who raised these ethical issues noted that the purpose of school education is not to train environmental scientists or experts in the globalisation of food systems. The messages needed to be informative and simple, but also limited. The importance of raising students’ basic awareness of ethical food-related issues was that it supports informed food choices among Australians.

### 3.4. Introductory Lessons about Foods from Farm to Plate to Make More Informed Food Choices

Most of the participants frequently reported that secondary school curricula needed to cover issues like: “where do foods come from?”, “how food is produced”, “what makes up a food product” and “which foods are manufactured”. Almost all of the interviewees reported that it was not appropriate at the secondary school-level to go into full details about foods journey from farm to plate. They made comments such as: “we have to be careful about the amount of details”; “really the basic stuff that kids don’t know is enough”; and “knowledge has to continue to building after people leave school”. A key purpose of secondary food curricula should be to enhance students’ basic understanding of current food production systems particularly in Australia and how the food production systems can affect human health. In addition, some of the professionals commented that raising high school students’ knowledge of current food production systems would enable students to develop a better understanding of the ethical issues linked to food production systems.

“If they eat bread then they need to know that that came from wheat and was made into flour and that was further processed and you know how it got to them.”

—Dietitian

## 4. Discussion

The findings reflected the perspectives of prominent food-related professionals in Australia regarding essential areas of nutrition and food systems (N&FS) for school-based education programs for Australian adolescents. Four essential areas emerged from the interview transcripts that reflected the broad role that food plays in young people’s lives and the breadth of knowledge and skills required: (1) Key nutrition messages to support a healthy lifestyle; (2) Skill development programs; (3) Food Ethics education; and, (4) Foods from farm to plate. Although the first and second areas are explored in previous studies [11,17,36], this investigation added new findings that are related to these two areas to be considered for secondary school education programs. Few studies have focused on the third and fourth areas of knowledge (Food Ethics education and Foods from farm to plate) for school-based food education programs (e.g., [24]). One reason for this gap is the primary focus of the majority of the previous reported studies on educating students about nutrition for better health outcomes [24]. The current study revealed that professionals held a more holistic view on N&FS education for secondary school students when compared to previous studies [21,24].

This study identified all these four areas as essential for secondary school education programs. However, the majority of previous studies have shown significantly stronger attention Key nutrition messages to support a healthy lifestyle and Food skills for better health outcomes among adolescents [11,17] as compared to the Food Ethics and Foods’ journey from farm to plate. Portraying these important areas together may provide new insights for curriculum developers in Australia to further assess the current gaps in secondary school curriculum.

The first identified area, ‘Key nutrition messages to support a healthy lifestyle’, mainly referred to the basic recommendations in the dietary guidelines, particularly in relation to the food group, is supported by previous literature [42,43,44]. However, the importance of educating senior high school students about nutrition requirements during pregnancy, identified in current study, has not been well addressed previously. The major focus of previous studies has been on nutrition education at the time or during the period of pregnancy [45,46], and not on preparation for pregnancy in the longer term. Recent Australian literature has identified the poor knowledge of pregnant women about important dietary recommendations [47]. The education of senior high-school students about the key nutrition requirements of pregnancy might positively impact the health status of young mums and their fetuses.

Continuing the theme of preparing for independent adult lives, the participants highlighted the need to create positive attitudes towards breastfeeding among senior secondary students. A study of women’s reasons for ceasing breast feeding [48] revealed that women who had the intention to breastfeed before delivery were more likely to initiate and continue breastfeeding when compared to those who did not plan or those who were unsure whether to breast feed [48]. The fostering of positive attitudes towards breastfeeding during adolescence might increase women’s intention to breast feed and men’s support for women who breast feed. Future school-based nutrition education programs targeting senior high-school students might consider this issue further.

The second identified area was the importance of development of food-related skills to enhance healthy dietary behaviours, which is aligned with previous studies [17,49]. The participants of the current study presented a broad refelection of essential food skills, such as critical or analytic approach to shopping, or informed participation in the food marketplace and its link to the interpreting of food label information to identify healthy food options. The participants also reported the application of this critical approach in the interpretation of food labels for ethical considerations as related to food production and procurement. Complementing this analytic approach was the identified need for practical skills, such as budgeting, growing food, and cooking, as supported by other studies [17,21,24]. Particular skills were identified as important, as well as the associated considerations around that skill, for example food preparation requires consideration of the food components, food safety, and nutrient content, which facilitate the process of communicating various important food topics with students and shows the efficacy of recommendations for everyday practices.

Notably, most of the participants in the present study placed stronger emphasis on food preparation, cooking, and food safety practices when compared to other food skills. A similar finding was reported by Ronto et al [49] in their study of home economics teachers’ views of food literacy. In addition, a recent review found a similar emphasis on food preparation skills [11]. In contrast to the review finding that most programs were directed towards younger adolescents, the present participants considered that these skills were necessary for all secondary school students with no preference toward younger adolescents.

The third identified area referred to the food ethics education. The current study provided a broad picture of important food-related ethical issues for secondary school education programs to support informed food choices among young Australian. There are very limited number of studies that referenced to the importance of ethic-based food related education within the school setting, such as those relating to animal welfare [36], environmental sustainability [25,36], and the supporting of local farmers [25,50]. In fact, the majority of investigations related to school-based education programs have greater focus on improving students’ health outcomes [24]. However, studies underlined the importance of increased consumer awareness of animal welfare to encourage the purchasing of animal welfare friendly food products [51,52,53] and increased awareness of food and environmental sustainability to promote the transition toward ecologically sustainable diets [28,29].

A recent study of adolescents’ food literacy in Australia showed that they have very limited knowledge of animal welfare and environmental sustainability issues [36]. However, another Australian study of 2204 adults showed that they were concerned about a variety of animal welfare and food-related environmental issues [29]. In contrast to our findings and to these two studies, a study related to Australia rated animal welfare and environmental sustainability as among the least important aspects of food literacy for secondary students from the perspectives of home economics teachers [49]. This might be linked to the nature of participants’ professions and the depths of their knowledge of these issues.

The last essential area identified in the present study concerned the journey of foods from farm to plate. Previous studies had considerably less focus on the exploration of school-based education programs that are related to food production systems and their impacts on health, environment, local farmers, and other issues when compared the first and second areas identified in this study. This might be due to the dominant focus of schools and nutrition researchers on the health-related aspect of nutrition for better health outcomes in children and adolescents [22,24]. However, literature have documented the strong links between food production systems and human health [28,54]; food production sectors and environmental sustainability [27,30]; and, food production sectors and animal welfare [51,53]. Correspondingly, the need to increase students’ awareness of these issues is reported in literature [24].

Overall, this study explored what experts considered to be essential knowledge areas that would facilitate informed food choice behaviours of young adults. A wide range of topics for food education programs of adolescents was identified as necessary, many of which were consistent with previous reported areas [17,24,26,36,49]. This study also identified other areas that are considered to be important for adolescents’ education programs that have been less reported in previous studies, for example, educating senior adolescents about nutrition requirements during pregnancy and the importance of creating positive attitude about breastfeeding. Other less explored areas identified were the non-health aspects of food systems education, including food production and a broad approach to food ethics.

These findings also might be helpful for other countries. A similar study conducted by the same authors in Iran [24] reported a considerable overlap with the range of N&FS areas.

Although nutrition knowledge is a key component of health literacy [55] and has been identified as essential for behaviour change [56], knowledge alone is not sufficient to change health-related practices [56]. Other influential factors include price, convenience [57], and the availability of healthy options [58] need to be considered.

## 5. Conclusions

This study provided a framework of essential nutrition issues in conjunction with important food system topics for secondary school education programs, comprising four main areas. The development of this multidimensional guide, which incorporated food systems and ethical issues into health-related aspects of foods, occurred through interviewing different groups of food professionals. Several components of the ‘Key nutrition messages to support a healthy lifestyle’ and ‘Skill development programs’ in the current study are supported in previous literature. However, this study also added new components to these areas. ‘Food Ethics education’ and ‘Foods from farm to plate’, identified in current study are significantly understimated in previous literature on school-based food education programs. Inclusion of all these four areas together in secondary school education programs may support the efficient interaction of the adolescents with food issues and may enable students to: make healthier food choices; to develop health promoting life skills; and, to make informed food choices that protect the environment, farm animal welfare, and local producers. Findings of this study may provide new insights for curriculum developers in Australia to further assess the strengths and gaps in N&FS components of the current secondary school curriculum.

The food professionals in this study were asked to express their opinion about essential N&FS knowledge issues for adolescents and were not asked to state their opinion regarding essential knowledge issues for children or adults more broadly. Future studies could target younger children’s N&FS education or N&FS components of primary school curriculum.

**Limitations:**

This study has contributed to one part of the picture of nutrition and food systems (N&FS) education in Australia. Other parts need to be identified by future studies beyond the professionals’ views of essential N&FS knowledge issues for adolescents. A systematic investigation of current N&FS components of school curriculum would be useful to specify the strengths and gaps regarding N&FS areas. How the current curriculum was developed and which groups of experts were involved in the development should be explored. It would also be very important to explore schoolteachers’ views regarding N&FS components of the school curriculum. At the same time, it is critical to identify how confident and informed schoolteachers are in educating adolescents about N&FS topics. Further areas for future research may also include: current knowledge of secondary school students of wide range of important N&FS issues; the average school hours that are currently allocated for N&FS education programs in secondary schools in Australia; and, identification of the maximum time that secondary schools’ curriculum in Australia can allocate for N&FS education.

Interviews commenced in 2012 and finished in 2014. A national school curriculum in Australia was introduced after the collection of data in this study and it is not clear the extent to which the participants’ issues have been addressed in the new curriculum, which is still in the implementation phase.

It would have been desirable to review and discuss the findings of this study with the participants. However, as reflected in the long period of time necessary to include all of the participants for data collection, the additional time required for such a review was not feasible. 

The variety of the recommendations of the participants and identified themes in this study largely depend on the nature of the participants’ professions. Broader sampling of other food professionals, for example agronomists and senior physical education teachers, may provide a broader set of knowledge and skill recommendations.

## Figures and Tables

**Table 1:** Summary of the ethical issues raised by the participants.

Key Food-Related Ethical Issue for Secondary School Curriculum	Example of Quotes
Simple animal welfare messages about the treatment of animals in food production systems	“School-leavers need to know about animal welfare aspect of food production. The way, which they are born, reared, handled, transported and killed for food. In terms of intensive food production like laid hens for eggs and meat, or pigs for meat they are done indoor”
	—Animal welfare expert
Globalization of the food supply and its impacts on the economy and the environment	“…the way that food is distributed, imported and exported. Impacts on the local businesses, local farmers. The role of industry and jobs and people that are involved and affected. A broad understanding in the community of the food system”
	—Public health expert
	“For instance the fashion to produce super foods likes chia and quinoa. The consequences of that in terms of economic costs, social costs both in the places of production and in the local communities”
	—Public health nutritionist
The environmental cost of global food transportation	“They need to know about the environmental cost of transport, like climate change. …I don’t think the education system either provides them with enough input”
	—Environmental scientists
The importance of supporting local farmers and small local businesses	“They need to know if they don’t buy Australian made and choose food, which is coming from overseas what implications that has then for the farmers in Australia…”
	—Dietitian
Sustainable agriculture vs. intensive agriculture	“I would like them to leave the school with some appreciation of sustainable agriculture not in great depth, but to know something about it”
	—Nutritionist
The effects of food wastage and food packaging on the environment	“Sharing the planet and having a responsible attitude towards food issues for example in terms of food wastage…also in relation to the environmental costs of over production…”
	—Public health nutritionist
Food security on global and community levels	“They need to understand food security on a global level and bringing it right down to a community level in terms of sort of the most vulnerable people in certain communities such as homeless, unemployed and those kinds of high risk groups. Some schools encourage children to do community service and that’s a good experience for them, perhaps if they can go to a soup kitchen or to volunteer. I think all school children should volunteer to deliver meals on wheels to older people because then it develops a healthy respect across the generation”
	—Public health nutritionist
